# Protocol for TRAUMADORNASE: a prospective, randomized, multicentre, double-blinded, placebo-controlled clinical trial of aerosolized dornase alfa to reduce the incidence of moderate-to-severe hypoxaemia in ventilated trauma patients

**DOI:** 10.1186/s13063-020-4141-6

**Published:** 2020-03-18

**Authors:** Julien Pottecher, Eric Noll, Marie Borel, Gérard Audibert, Sébastien Gette, Christian Meyer, Elisabeth Gaertner, Vincent Legros, Raphaël Carapito, Béatrice Uring-Lambert, Erik Sauleau, Walter G. Land, Seiamak Bahram, Alain Meyer, Bernard Geny, Pierre Diemunsch

**Affiliations:** 1 Hôpitaux Universitaires de Strasbourg, Hôpital de Hautepierre, Service d’Anesthésie-Réanimation Chirurgicale, 1 Avenue Molière, 67098 Strasbourg, France; 2 Université de Strasbourg, Faculté de Médecine, Fédération de Médecine Translationnelle de Strasbourg (FMTS), EA3072, 4 Rue Kirschleger, 67085 Strasbourg, France; 3 Fédération Hospitalo-Universitaire OMICARE, Centre de Recherche d’Immunologie et d’Hématologie, 4 rue Kirschleger, 67085 Strasbourg Cedex, France; 4 Sorbonne Universités, UPMC Université Paris 06, INSERM UMR_S 1158 Neurophysiologie Respiratoire Expérimentale et Clinique, AP-HP, Groupe Hospitalier Pitié-Salpêtrière Charles Foix, Département d’Anesthésie Réanimation, 47-83 Boulevard de l’Hôpital, 75651 Paris Cedex 13, France; 5 CHRU Nancy, Hôpital Central, Service d’Anesthésie-Réanimation, 29 Avenue de Lattre de Tassigny, 54000 Nancy, France; 6 CHR Metz-Thionville—Site de Mercy, Service de Réanimation Polyvalente, 1 Allée du Château, 57350 Ars-Laquenexy, France; 7 Groupe Hospitalier de la Région de Mulhouse et Sud Alsace (GHRMSA), Pôle d’Anesthésie-Réanimation, 20 rue du Dr Laennec, 68051 Mulhouse Cedex 1, France; 8 Hôpital Louis Pasteur, Service d’Anesthésie-Réanimation Pôle 2, 39 Avenue de la Liberté, 68024 Colmar Cedex, France; 9 CHU de Reims, Hôpital Maison Blanche, Réanimation Chirurgicale et Traumatologique, SAMU 51, 45 rue Cognacq-Jay, 51092 Reims, France; 10 Hôpitaux Universitaires de Strasbourg, Nouvel Hôpital Civil, Laboratoire Central d’Immunologie, 1 Place de l’Hôpital, 67091 Strasbourg Cedex, France; 11 Université de Strasbourg, Faculté de Médecine, Fédération de Médecine Translationnelle de Strasbourg (FMTS), Laboratoire d’ImmunoRhumatologie Moléculaire, INSERM UMR_S 1109, 4 rue Kirschleger, 67085 Strasbourg Cedex, France; 12 Hôpitaux Universitaires de Strasbourg, Hôpital Civil, Pôle Santé Publique, Groupe Méthode en Recherche Clinique (GMRC), 1 Place de l’Hôpital, 67091 Strasbourg Cedex, France; 13 Hôpitaux Universitaires de Strasbourg, Nouvel Hôpital Civil, Service de Physiologie et d’Explorations Fonctionnelles, 1 Place de l’Hôpital, 67091 Strasbourg Cedex, France

**Keywords:** Acute respiratory distress syndrome, Adult, Hypoxaemia, Multiple trauma, Deoxyribonuclease I, Neutrophil extracellular traps, Randomized controlled trial

## Abstract

**Background:**

Acute respiratory distress syndrome continues to drive significant morbidity and mortality after severe trauma. The incidence of trauma-induced, moderate-to-severe hypoxaemia, according to the Berlin definition, could be as high as 45%. Its pathophysiology includes the release of damage-associated molecular patterns (DAMPs), which propagate tissue injuries by triggering neutrophil extracellular traps (NETs). NETs include a DNA backbone coated with cytoplasmic proteins, which drive pulmonary cytotoxic effects. The structure of NETs and many DAMPs includes double-stranded DNA, which prevents their neutralization by plasma. Dornase alfa is a US Food and Drug Administration-approved recombinant DNase, which cleaves extracellular DNA and may therefore break up the backbone of NETs and DAMPs. Aerosolized dornase alfa was shown to reduce trauma-induced lung injury in experimental models and to improve arterial oxygenation in ventilated patients.

**Methods:**

TRAUMADORNASE will be an institution-led, multicentre, double-blinded, placebo-controlled randomized trial in ventilated trauma patients. The primary trial objective is to demonstrate a reduction in the incidence of moderate-to-severe hypoxaemia in severe trauma patients during the first 7 days from 45% to 30% by providing aerosolized dornase alfa as compared to placebo. The secondary objectives are to demonstrate an improvement in lung function and a reduction in morbidity and mortality. Randomization of 250 patients per treatment arm will be carried out through a secure, web-based system. Statistical analyses will include a descriptive step and an inferential step using fully Bayesian techniques. The study was approved by both the Agence Nationale de la Sécurité du Médicament et des Produits de Santé (ANSM, on 5 October 2018) and a National Institutional Review Board (CPP, on 6 November 2018). Participant recruitment began in March 2019. Results will be published in international peer-reviewed medical journals.

**Discussion:**

If early administration of inhaled dornase alfa actually reduces the incidence of moderate-to-severe hypoxaemia in patients with severe trauma, this new therapeutic strategy may be easily implemented in many clinical trauma care settings. This treatment may facilitate ventilator weaning, reduce the burden of trauma-induced lung inflammation and facilitate recovery and rehabilitation in severe trauma patients.

**Trial registration:**

ClinicalTrials.gov, NCT03368092. Registered on 11 December 2017.

## Administrative information

Note: the numbers in curly brackets in this protocol refer to SPIRIT checklist item numbers. The order of the items has been modified to group similar items (see http://www.equator-network.org/reporting-guidelines/spirit-2013-statement-defining-standard-protocol-items-for-clinical-trials/).

**Table:** 

Title {1}	Protocol for TRAUMADORNASE: a prospective, randomised, multicentre, double-blinded, placebo-controlled clinical trial of aerosolized dornase alfa to reduce the incidence of moderate to severe hypoxemia in ventilated trauma patients
Trial registration {2a and 2b}	ClinicalTrials.gov NCT03368092
Protocol version {3}	8.0, November 13, 2018
Funding {4}	Aerogen (Ireland) will provide nebulizers to study centres at an estimated value of 28k€. After validation from its Scientific Committee, the TRAUMADORNASE study is supported by a 300k€ grant from the French Ministry of Health (PHRCI-2017-S09).
Author details {5a}	^1^ Hôpitaux Universitaires de Strasbourg, Hôpital de Hautepierre, Service d’Anesthésie-Réanimation Chirurgicale, 1 Avenue Molière, 67098 Strasbourg, France. ^2^ Université de Strasbourg, Faculté de Médecine, Fédération de Médecine Translationnelle de Strasbourg (FMTS), EA3072, 4 Rue Kirschleger, 67085 Strasbourg, France. ^3^ Fédération Hospitalo-Universitaire OMICARE, Centre de Recherche d’Immunologie et d’Hématologie, 4 rue Kirschleger, 67085 Strasbourg Cedex, France. ^4^ Sorbonne Universités, UPMC Université Paris 06, INSERM UMR_S 1158 Neurophysiologie Respiratoire Expérimentale et Clinique, AP-HP, Groupe Hospitalier Pitié-Salpêtrière Charles Foix, Département d'Anesthésie Réanimation, 47-83 Boulevard de l’Hôpital 75651 Paris Cedex 13, France. ^5^ CHRU Nancy, Hôpital Central, Service d’Anesthésie-Réanimation, 29 Avenue de Lattre de Tassigny, 54000 Nancy, France. ^6^ CHR Metz-Thionville – Site de Mercy, Service de Réanimation Polyvalente, 1 Allée du Château, 57350 Ars Laqueney, France ^7^ Groupe Hospitalier de la Région de Mulhouse et Sud Alsace (GHRMSA), Pôle d’Anesthésie-Réanimation, 20 rue du Dr Laennec, 68051, Mulhouse Cedex 1, France. ^8^ Hôpital Louis Pasteur, Service d’Anesthésie-Réanimation Pôle 2, 39 Avenue de la Liberté, 68024 Colmar Cedex, France. ^9^ CHU de Reims, Hôpital Maison Blanche, Réanimation Chirurgicale et Traumatologique – SAMU 51, 45 rue Cognacq-Jay 51092, Reims, France. ^10^ Hôpitaux Universitaires de Strasbourg, Nouvel Hôpital Civil, Laboratoire Central d’Immunologie, 1 Place de l’Hôpital 67091 Strasbourg Cedex, France. ^11^ Université de Strasbourg, Faculté de Médecine, Fédération de Médecine Translationnelle de Strasbourg (FMTS), Laboratoire d’ImmunoRhumatologie Moléculaire, INSERM UMR_S 1109, 4 rue Kirschleger, 67085 Strasbourg Cedex, France. ^12^ Hôpitaux Universitaires de Strasbourg, Hôpital Civil, Pôle Santé Publique, Groupe Méthode en Recherche Clinique (GMRC), 1 Place de l’Hôpital, 67091 Strasbourg Cedex, France. ^13^ Hôpitaux Universitaires de Strasbourg, Nouvel Hôpital Civil, Service de Physiologie et d’Explorations Fonctionnelles, 1 Place de l’Hôpital 67091 Strasbourg Cedex, France.
Name and contact information for the trial sponsor {5b}	French Ministry of Health, GIRCI Est, Ms Nathalie PORTIER, CHU de Dijon, 14, rue Paul Gaffarel, BP 77908, 21079 Dijon Cedex, France
Role of sponsor {5c}	Funders will have no role in the study’s design, collection, management, analysis, interpretation of data, writing of the report, and the decision to submit the report for publication.

## Introduction

### Background and rationale {6a}

Severe trauma remains a major socio-economic burden worldwide [1, 2]. Indeed, it is the third cause of fatality overall, the first cause of fatality and invalidity in the 16–45 age group and the first cause of disability-adjusted life years (DALYs). Aside from civilian and military trauma cases, terrorist attacks have added new threats [3]. While the first peak of trauma-associated mortality happens within the very first hours from exsanguination and severe central nervous system injuries, secondary deaths are triggered by multi-organ failure (MOF) and acute respiratory distress syndrome (ARDS) in the intensive care unit (ICU) [4].

The taxonomy of ARDS was recently refined by the last Berlin definition [5], which also distinguished three levels of increasing hypoxaemia severity (mild/moderate/severe) based on the ratio of partial arterial oxygen tension (PaO_2_) over inspired oxygen fraction (FiO_2_). Patients who develop moderate-to-severe ARDS in the ICU have a worse prognosis compared to mild ARDS patients, including increased mortality rates (48% vs*.* 29%), impaired functional recovery, compromised quality of life and cognitive dysfunction [6]. Severe trauma definitely remains a significant risk factor for hypoxaemia, implicating both direct and indirect lung injuries [7]. Notwithstanding improvements in prehospital care, resuscitation and mechanical ventilation, the incidence of hypoxaemia in trauma patients has remained consistently high during the last 30 years [8–11]. In the most severely injured trauma patients (Injury Severity Score (ISS) [12] above 15) requiring blood transfusion, the incidence of hypoxaemia may exceed 40%. Indeed, a recent analysis of the PROMMTT registry underlines that the incidence of moderate-to-severe hypoxaemia could be as high as 45% [13]. In trauma patients, ARDS increases the duration of mechanical ventilation, ICU and hospital lengths of stay, incidence of ventilation-acquired pneumonias, healthcare-associated costs and mortality [14].

### Pathophysiology of trauma-associated hypoxaemia and acute respiratory distress syndrome

As previously stated [7], severe trauma may contribute to hypoxaemia by both direct injuries (lung contusion, aspiration) and indirect injuries (non-thoracic trauma, musculoskeletal injuries, haemorrhagic shock, transfusion-associated acute lung injury [15, 16]). Whatever the mechanism implicated, inflammation is a key player [17–19]. Indeed, tissue injury triggers a massive and short-lived release of damage-associated molecular patterns (DAMPs) [20], which bind both Toll-like receptors (TLRs) [21] and receptors for advanced glycation end products (RAGE) [22, 23], which recruit and activate neutrophils, resulting in a widespread systemic inflammatory response [24].

The molecular structure of DAMPs is diverse but the most potent are made of double-stranded DNA [25], either fully (e.g. mitochondrial DNA [26–28]) or partly (e.g. nucleosomes [29], high mobility group box-1 (HMGB1), heat shock proteins (HSP)).

Once bound to neutrophils, DAMPs induce profound conformational changes in these cells (NETosis), which trigger both non-self pathogen killing [30] and self tissue injury [22, 31]. Indeed, NETosis refers to the release of neutrophil extracellular traps (NETs), composed of a backbone (decondensed chromatin fibres) coated with antimicrobial granular and cytoplasmic proteins, such as myeloperoxidase, neutrophil elastase (NE) and α-defensins [32, 33]. The detrimental effects of excessive NET release are particularly important to ARDS, because NETs can expand more easily in the pulmonary alveoli, causing extensive lung injury [33] and hypoxaemia. Moreover, while unbound NE is usually rapidly inactivated when released into plasma, DNA-bound NE is protected from neutralization by plasma [34].

Double-stranded DNA thus constitutes the backbone of both DAMPs and NETs, and prevents NETs from plasma neutralization. Extracellular DNA is physiologically broken up by endogenous deoxyribonucleases (DNases [33, 35]), which may become overwhelmed by a massive influx of both DAMPs and NETs. This is exacerbated as the activity of endogenous DNases is reduced in severe trauma patients (0.059 ± 0.0033 U/ml) compared to healthy controls (0.174 ± 0.031 U/ml; *p* < 0.0001 [36]).

However, an FDA-approved recombinant DNase has been commercially available since 1994 (dornase alfa, Pulmozyme; Roche, Basel, Switzerland and Genentech, San Francisco, CA, USA) and prescribed for the treatment of pulmonary exacerbations in cystic fibrosis patients. As dornase alfa is usually administered via the intratracheal route (aerosols), its biological actions and pharmacokinetic properties could be an excellent prerequisite for a clinical breakthrough in trauma-induced hypoxaemia. Indeed, dornase alfa was shown to reduce trauma-induced lung injury in mice [37], to fight against sepsis-induced ARDS [38, 39] and to reduce mechanical ventilation-induced lung injury [40], which are traditional “second hits” for lung damage in ventilated trauma patients. In a small, randomized clinical trial, aerosolized dornase alfa was also shown to improve oxygenation in mechanically ventilated ICU patients with lobar atelectasis [41].

### Objectives {7}

#### Primary objective

The primary objective of the TRAUMADORNASE study is to demonstrate a reduction in the incidence of moderate-to-severe hypoxaemia from 45% to 30% in severe trauma patients during the first 7 ICU days by providing aerosolized dornase alfa once during the first 2 ICU days as compared to equivalent provision of placebo (NaCl 0.9%).

#### Secondary objectives

The secondary objectives are to demonstrate, using aerosolized dornase alfa as compared to placebo, an improvement in static lung compliance, a reduction in mechanical ventilation duration or an increase in ventilation-free ICU days, a reduction in the length of ICU stay, a reduction in the hospital length of stay, a reduction in the incidence of multi-organ failure, a reduction in the incidence of ventilator-associated pneumonia (VAP) and a reduction in mortality at day 30.

### Trial design {8}

This will be an investigator-initiated, institution-led, multicentre, double-blinded, placebo-controlled, parallel-group, superiority, randomized trial in ventilated, trauma ICU patients.

Randomization will be carried out through a secure web-based randomization system, stratified by the centre and the presence of severe traumatic brain injury (Glasgow Coma Score < 9 on scene).

## Methods: participants, interventions and outcomes

### Study setting {9}

The study will be conducted in seven French participating hospitals, both university-affiliated and non-university-affiliated.

### Eligibility criteria {10}

Inclusion criteria will be checked before inclusion in the study.

The inclusion criteria are as follows:

- Adult patient (> 18 years old) of either sex affiliated to the National Health Service
- Severe trauma patient (either blunt or penetrating) with ISS [12] > 15
- Under mechanical ventilation for an expected duration > 48 h
- Admitted to the ICU
- Signed informed consent from the patient or the patient’s relative or emergency consent procedure
- Patient equipped with an indwelling arterial catheter
- Negative pregnancy test in women of childbearing age

The exclusion criteria are as follows:

- Pregnancy or breast-feeding
- Opposition from the patient or his/her relatives
- Protected major (Guardianship)
- Contraindication to the use of dornase alfa
- Known intolerance to dornase alfa
- Patient whose life expectancy is less than 24 h, according to the treating physician
- “Do not resuscitate” order

### Who will take informed consent? {26a}

Inclusion will be feasible after patient approval, relative approval or emergency consent procedure (according to French law [42]).

Subsequent confirmation of consent will be obtained from the relatives and from the patient as soon as possible. The consent forms are available from the corresponding author on request.

After primary haemostasis and emergent surgical interventions, patients will be randomized in the ICU within 6 h. In the case of emergent surgical intervention before ICU admission, a maximum delay of 18 h will be tolerated from hospital admission (trauma bay) to study drug administration. Day 0 will be considered the day of ICU admission.

### Additional consent provisions for collection and use of participant data and biological specimens {26b}

Additional consent will be required for the collection of biological specimens in ancillary studies, which will be stored for a maximum duration of 15 years.

### Interventions

#### Explanation for the choice of comparators {6b}

The comparator will be normal saline (NaCl 0.9%, 2.5 ml, administered through the Aerogen solo device). NaCl is neutral regarding DAMPs, NETs and occurrence of either hypoxaemia or ARDS, and therefore is considered a placebo.

#### Intervention description {11a}

Treatment with either dornase alfa or placebo will be administered using aerosol (Aerogen solo) in the ventilation circuit once per day (average treatment length: 7 min) for the first 2 days. The Aerogen device was shown to optimize dornase alfa deposition in the distal lung airways [43, 44]. Dornase alfa has an excellent safety profile and aerosolized NaCl 0.9% has a neutral effect on lung physiology.

The variables under study will be gathered every day and recorded on the electronic clinical research form (CleanWeb; Telemedicine Technologies S.A.S., Boulogne Billancourt, France).

For safety purposes, patient variables will be closely monitored before, during and within the first post-administration hour: lowest SpO_2_, maximal value and maximal increase in peak inspiratory airway pressure, maximal value and maximal increase in plateau airway pressure, extreme values of heart rate and mean arterial pressure, skin erythema, urticaria and variations in central temperature exceeding 1 °C.

During the first 7 days, at least one blood gas analysis and chest X-ray will be performed every day at 8:00 a.m. to compute the primary endpoint: presence or absence of ARDS and severity of hypoxaemia according to the Berlin definition. Additional blood gas analysis will be allowed and the worst daily PaO_2_/FiO_2_ ratio will be considered.

#### Ancillary mechanistic study

On days 0, 3 and 5, additional blood samples (6 ml on each day) will be drawn into EDTA tubes, centrifuged and stored (− 80 °C) for subsequent analysis of DAMPs (mitochondrial DNA by qPCR; HMGB1, HSP70 and sRAGE by ELISA) at the end of enrolment. Whole blood samples will be drawn (days 0, 3 and 5) for extemporaneous quantification of NETs on fresh blood using a flow cytometric assay [45] in patients at the Strasbourg centre.

### Criteria for discontinuing or modifying allocated interventions {11b}

In the case of an adverse event following treatment administration (desaturation, bronchospasm, anaphylactic reaction), treatment will be immediately discontinued and the second treatment dose will not be given on day 1.

### Strategies to improve adherence to interventions {11c}

In each centre, boxes containing both full and empty treatment vials will be returned to the pharmacy responsible for clinical studies. For every included patient, a sheet will be completed (date, hour, nurse in charge) and signed for every study treatment preparation, administration and clinical surveillance.

For safety purposes, patient variables will be closely monitored before, during and within the first post-administration hour: lowest SpO_2_, maximal value and maximal increase in peak inspiratory airway pressure, maximal value and maximal increase in plateau airway pressure, extreme values of heart rate and mean arterial pressure, skin erythema, urticaria and variations in central temperature exceeding 1 °C.

At least one blood gas analysis and chest X-ray will be performed every day at 8:00 a.m. to compute the primary endpoint: presence or absence of ARDS and severity of hypoxaemia according to the Berlin definition. Additional blood gas analysis will be allowed and the worst daily PaO_2_/FiO_2_ ratio will be taken into account.

### Relevant concomitant care permitted or prohibited during the trial {11d}

#### Standardization of respiratory care

Daily care for the included patients will be protocolized according to good clinical practices, especially concerning respiratory care (semi-recumbent position, protective mechanical ventilation (6–8 ml/kg predicted body weight), PEEP > 5 cmH_2_O, plateau pressure < 30 cmH_2_O, close tracheal cuff pressure monitoring, early enteral feeding (500 ml on day 1), glucose control and protocolized sedation based on both CPOT and RASS scores [46]). Adherence to guidelines will be checked in every centre for every patient.

Patients will be followed until day 30 for the record of study outcomes.

Every concomitant care will be allowed except aerosols during study drug administration.

### Provisions for post-trial care {30}

Post-trial care is not planned. Patients who suffer harm from trial participation will be cared for in the intensive care unit. Should prejudice linked to study participation occur, financial compensation will be provided by the insurance (Société Hospitalière d’Assurances Mutuelles—SHAM, 18 rue Edouard Rochet, 69,372 Lyon Cedex 08, France; contract number: 143.380) contracted by the promotor (Hôpitaux Universitaires de Strasbourg).

At 6 months, the respiratory status will be assessed using the modified MRC dyspnoea questionnaire [47, 48] and a chest X-ray.

### Outcomes {12}

#### Primary endpoint

The primary endpoint will be the incidence of moderate-to-severe hypoxaemia (PaO_2_/FiO_2_ < 200, according to the Berlin definition [5]) in severe trauma patients (ISS > 15) during the first 7 ICU days. The PaO_2_/FiO_2_ ratio will be computed at least once daily (8:00 a.m.) together with the supine chest X-ray and the worst daily PaO_2_/FiO_2_ value will be taken into account to define hypoxaemia severity. In ARDS patients, the severity of hypoxaemia allows for its classification according to the Berlin definition and is strongly associated with mortality, length of recovery and quality of life [6, 7].

#### Secondary endpoints

The following secondary endpoints will be recorded:

- Static lung compliance (ml/cmH_2_O) (measured at least once daily at 8:00 a.m. during the first 7 days)
- Duration of mechanical ventilation (h) from ICU admission to first extubation success (> 48 h without reintubation)
- Length of ICU stay (h)
- Length of stay in the hospital (days)
- Incidence of multi-organ failure (a SOFA score of 3 or more in at least two organ systems [49]), assessed daily during the first 7 days
- Incidence of VAP according to both the American Thoracic Society (ATS) [50] and the Center for Disease Control and Prevention (CDC) [51] definitions, assessed daily during the first 7 days
- Mortality on day 30

The effects of dornase alfa and normal saline will be assessed according to the plasma concentrations of DAMPs (mitochondrial DNA, HMGB-1, HSP70, sRAGE) and NETs (Strasbourg centre only) divided into quartiles at day 0, day 3 and day 5. It is anticipated that trauma patients with the highest blood concentrations of either DAMPs or NETs will develop the most severe complications (including hypoxaemia and ARDS). The time course of DAMP and NET blood concentrations will also be analysed according to treatment group to unveil a potential quicker decrease in patients randomized in the dornase alfa group.

### Participant timeline {13}

The total duration of participation in the study will be 30 days. The forecast study duration is 36 months from first to last patient recruitment (Table 1).

**Table 1:** Template of recommended content for the schedule of enrolment, interventions and assessments

	Visit				
	Information	Enrolment	Allocation, visit 0	Post-allocation, visits 1–7	Close-out
Timepoint	Day 0	Day 0	Day 0	Days 1–7	Day 30
Location	Hospital	Hospital	Hospital	Hospital	Hospital
Next of kin information	X*	X*	X		
Signed informed consent		X*			
Plasma **β**HCG assay		X(*)			
Eligibility criteria check	X*	X*	X*		
Randomization			X*		
Study drug administration			X*^d^	X*^d^	
Clinical examination^a^		X	X	X	
Diagnostic tests^b^		X	X	X	
Blood assay^c^			X*	X*	
Adverse effect recording		X*	X*	X*	X*
Concomitant medications		X	X	X	X

*βHCG* beta human chorionic gonadotropin, *X* diagnostic test performed on a daily basis according to the standard of care in participating centres, X* diagnostic tests performed for the purpose of the TRAUMADORNASE study

^a^Clinical examination includes physical examination (auscultation of the chest, central body temperature, positive end-expiratory pressure and inspired oxygen fraction levels) and recording of Utstein criteria [49]

^b^Diagnostic tests include arterial blood gases, chest X-ray, leukocyte and platelet counts, creatinine, blood urea nitrogen, bilirubin and quantitative lung bacteriologic samplings (bronchoalveolar lavage fluid or protected specimen brush) in the case of suspected lung infection

^c^Blood withdrawal: 6 ml of blood on day 0, day 3 and day 5

^d^Study treatment will be given on day 0 and day 1

### Sample size {14}

The sample size was determined to be 250 subjects per arm (i.e. 500 subjects in total). Dornase alfa is expected to reduce the incidence of moderate-to-severe hypoxaemia from 0.45 to 0.30. Considering a reasonable standard deviation of 0.03, and using Bayesian techniques [52], 200 subjects per arm were estimated to show a difference of more than 0.12 (instead of the expected 0.15). Assuming 25% loss to follow-up, this number was increased to 250 subjects per arm, although these subjects will not be replaced.

### Recruitment {15}

Patients will be recruited in seven French participating hospitals, both university-affiliated and non-university-affiliated and admitting severe trauma patients:

- Hôpitaux Universitaires de Strasbourg, Hôpital de Hautepierre (150 severe trauma patients per year)
- Hôpitaux de Colmar, Hôpital Louis Pasteur (50 severe trauma patients per year)
- Centre Hospitalo-Universitaire de Reims, Hôpital Maison Blanche (150 severe trauma patients per year)
- Centre Hospitalier Régional Metz-Thionville, Hôpital de Mercy (50 severe trauma patients per year)
- Groupe Hospitalier de la Région de Mulhouse et Sud-Alsace, Hôpital du Moenchsberg—Emile Muller (50 severe trauma patients per year)
- Centre Hospitalier Régional Universitaire de Nancy, Hôpital Central (220 severe trauma patients per year)
- Assistance Publique—Hôpitaux de Paris, Hôpital Universitaire Pitié Salpêtrière—Charles Foix (500 severe trauma patients per year)

Taken as a whole, more than 3500 patients per year fulfil the inclusion criteria, allowing for an inclusion ratio of one patient included out of seven patients admitted to one of the participating centres.

### Assignment of interventions: allocation

#### Sequence generation {16a}

Randomization will be conducted over a dedicated, password-protected, SSL-encrypted website (CleanWeb; Telemedicine Technologies S.A.S.) to allow immediate and concealed allocation. Allocation will also be stratified by centre and the presence of severe traumatic brain injury (Glasgow Coma Score < 9 on scene).

#### Concealment mechanism {16b}

The experimental study drug and placebo will be provided in identical boxes, allowing double-blind administration. The logistics of the trial fluid distribution to each of the seven participating centres that are anticipated to be recruiting will be coordinated by the pharmacy of the coordinating centre (Hôpitaux Universitaires de Strasbourg).

#### Implementation {16c}

The allocation sequence will be computer-generated (CleanWeb; Telemedicine Technologies S.A.S.). Patients will be enrolled by registered investigators, who will also assign patients to a treatment consisting of either dornase alfa or placebo.

### Assignment of interventions: blinding

#### Who will be blinded {17a}

Trial participants, care providers, outcome assessors and data analysts will remain blinded after assignment to interventions, until the final analysis.

#### Procedure for unblinding if needed {17b}

Unblinding is permissible whenever an adverse event occurs, via immediate request to the poison centre of the study coordinator hospital (Hôpitaux Universitaires de Strasbourg) 24 h per day and 365 days per year. The procedure for revealing a participant’s allocated intervention during the trial includes an explicit mention in the patient record.

### Data collection and management

#### Plans for assessment and collection of outcomes {18a}

Clinical research associates will ensure that patient inclusion, data collection, registry and rapport are in line with the protocol, and that the study is conducted in accordance with the Good Clinical Practice guidelines. Furthermore, clinical research associates will check the following variables: patient initials, date of birth, sex, signed consent form, eligibility criteria, date of randomization, treatment assignment, adverse events and study endpoints. The data monitoring committee is institution-based and independent from potential industrial sponsors.

#### Plans to promote participant retention and complete follow-up {18b}

A dedicated card will be given to any included patient and participation in the TRAUMADORNASE trial will be explicitly mentioned during transfer to another ward or hospital during handovers.

#### Data management {19}

Data will be collected in each centre by clinical data technicians on an electronic case report form (CleanWeb; Telemedicine Technologies S.A.S.) using double password-protected computers. Pre-specified lists, range of values and drop-down menus in the electronic case report form will facilitate data entry and prevent writing errors. Study documents will be de-identified, stored in each recruitment centre and kept for at least 15 years in a locked, secure office, according to French law. All personnel involved in data analysis will be masked. Only the principal investigators and the statisticians will have access to the final data set.

#### Confidentiality {27}

People with direct access to the data will take all necessary precautions to maintain confidentiality. All data collected during the study will be rendered anonymous. Only initials and inclusion number will be registered.

#### Plans for collection, laboratory evaluation and storage of biological specimens for genetic or molecular analysis in this trial/future use {33}

On days 0, 3 and 5, additional blood samples (6 ml on each day) will be drawn into EDTA tubes, centrifuged and stored (− 80 °C) for subsequent analysis of DAMPs (mitochondrial DNA by qPCR; HMGB1, HSP70 and sRAGE by ELISA) at the end of enrolment. In patients included in the Strasbourg centre, whole blood samples will be drawn (days 0, 3 and 5) for extemporaneous quantification of NETs on fresh blood using a flow cytometric assay [45]. The remaining biological specimens will be stored in 0.5-ml aliquots at “Biomax” biobank, Laboratoire d’ImmunoRhumatologie Moléculaire, INSERM UMR_S 1109, 4, rue Kirchleger, 67,085 Strasbourg, France (Pr Siamak Bahram) for a maximum duration of 15 years (BB-0033-00084, CIM code: I00-I99 / A00-B99 / D50-D89 / M00-M99 / F00-F99 / G00-G99).

### Statistical methods

#### Statistical methods for primary and secondary outcomes {20a}

Statistical analyses will include a descriptive step and an inferential step using fully Bayesian techniques. The estimates will use Markov chains to Monte Carlo integrations (McMC), choosing prior distributions to be nearly conjugated situations. Unless the diagnoses for convergence give clues to the contrary, we will use three Markov chains with separated starting points, a burn-in of 100,000 for each chain and 100,000 more iterations with a thinning of 25 for building a total sample of 12,000 iterations on which the Monte Carlo integrations are used to retrieve characteristics of posterior distributions. The analyses will be carried out using R software (with ad hoc packages) and OpenBUGS. Sensitivity analyses will be systematically conducted, considering three scenarios with different priors: a default non-informative prior (e.g. Jeffreys prior), then an optimist prior and, finally, a pessimist prior.

In the descriptive step, all of the variables collected will be summarized: number and frequency for qualitative variables (ordinal and categorical) and minimum, quantiles (2.5, 25, 50, 75 and 97.5), maximum, mean and standard deviation for quantitative variables (discrete and continuous). For variables gathered over time, these descriptions will be provided globally and at each time. This description will be enriched by inference to extrapolate the observed quantities on the sample. For quantitative variables, we will assume a normal likelihood combined with a normal prior on the mean (mean 0 and variance 100) and γ on the precision (inverse of variance) with parameters 0.0005 and 0.005, and therefore mean 0.1 and variance 20. For binary variables (for which one proportion needs to be estimated), we will assume a binomial likelihood and a β prior on the proportion (Jeffreys prior with parameters 0.5 and 0.5, and thus mean 0.5 and variance 0.125). For categorical variables with more than two categories, we will assume a categorical likelihood together with a Dirichlet prior (Jeffreys prior with all parameters at 0.5).

The aim of this study is to show that the frequency of moderate-to-severe hypoxaemia is lower in the dornase alpha group than in the placebo group. The main variable is then dichotomous “moderate-to-severe hypoxaemia yes/no”, modelled in a logistic mixed regression. We will assume that this variable is Bernoulli distributed with parameter π. The logit of this parameter (linear predictor) is additively written as:

$$ \mathrm{logit}\left({\pi}_i\right)={\alpha}_0+{\alpha}_1I\left({g}_i=1\right)+{\beta}_i, $$

where:

- *α*_0_ is a grand mean, with mean 0 normal prior (the variance in this normal is 6, corresponding to a low informative prior)
- *I*(*g*_*i*_ = 1) is a dummy covariate coded for the group of subject *i* (1 for the dornase alpha group and 0 for the placebo group) — the prior on the parameter of this covariate is the same normal as that for the grand mean
- *β*_*i*_ is a (random) subject effect, on which is assumed a normal prior with mean 0 and low variance (e.g. 10)

Because the linear predictor is on the logit scale, the probability for moderate-to-severe hypoxaemia will be obtained by monitoring the back-transformation of the logit.

This regression model without covariates except group will be completed for taking into account potential confounding variables. In the model, the entire set of these variables will be added and, secondly, selected using stochastic search variable selection (SSVS) [53]. In such a model, the prior distribution on each parameter is a mixture of two mean 0 normal distributions, one with low variance and the other with high variance: if the posterior weight on this second normal is strongly around 0, then the prior on the parameter is essentially driven by a normal distribution whose mean is centred on 0; this is the clue for a “non-significant” parameter.

The secondary analyses will be conducted as the main analysis with a regressive model, testing the difference of a parameter between the two groups. Only the likelihood model will be changed to take into account the type of variable studied: γ distribution for continuous variables such as length of stay and duration of ventilation. Dichotomous variables such as 30-day mortality will be studied with logistic regression.

No statistical procedure for replacing missing values will be used. All variables and subjects will be considered in the descriptive analyses, but, for inference, 20% missing data or more will result in rejection of the variable or individual.

#### Interim analyses {21b}

An interim analysis will be performed after inclusion of the first 250 patients. These preliminary data will be available to the data safety and monitoring board (see later for details), which will have the ability to stop the trial for either futility or harm.

#### Methods for additional analyses (e.g. subgroup analyses) {20b}

Analyses will be performed in intention to treat. To verify the impact of possible deviations from the protocol, these analyses will be supplemented by an analysis per protocol. Subgroup analyses will be conducted according to the Glasgow Coma Scale on site (score either ≤ 8 or > 9).

#### Methods in analysis to handle protocol non-adherence and any statistical methods to handle missing data {20c}

No statistical procedure for replacing missing values will be used. All variables and subjects will be considered in the descriptive analyses, but, for inference, 20% missing data or more will result in rejection of the variable or individual.

#### Plans to give access to the full protocol, participant-level data and statistical code {31c}

The protocol is available on the ClinicalTrials.gov website (https://clinicaltrials.gov/ct2/show/NCT03368092?term=traumadornase&draw=2&rank=1). Study documents will be de-identified, stored in each recruitment centre and kept for at least 15 years in a locked, secure office, according to French law. All personnel involved in data analysis will be masked. Only the principal investigators and the statisticians will have access to the final data set.

### Oversight and monitoring

#### Composition of the coordinating centre and trial steering committee {5d}

Coordinating centre: Direction de la Recherche Clinique et de l’Innovation (DRCI) des Hôpitaux Universitaires de Strasbourg, 1 Place de l’Hôpital, 67,085 Strasbourg Cedex, France.

Steering committee: Julien Pottecher, MD, PhD (Hôpitaux Universitaires de Strasbourg), Eric Noll, MD, PhD (Hôpitaux Universitaires de Strasbourg), Mathieu Raux, MD, PhD (AP-HP, Hôpital Universitaire Pitié Salpêtrière), Gérard Audibert, MD, PhD (CHRU Nancy), Alain Leon, MD, PhD (CHU de Reims) and Pierre Diemunsch, MD, PhD (Hôpitaux Universitaires de Strasbourg).

Endpoint adjudication committee: TraumaBase Group.

Data management team: Erik Sauleau, MD, PhD and Groupe Méthode en Recherche Clinique (GMRC), Hôpitaux Universitaires de Strasbourg, Strasbourg, France.

#### Composition of the data monitoring committee, its role and reporting structure {21a}

The data safety and monitoring board (DSMB) will include Dr Laure Peyro-Saint Paul (drug monitoring specialist), Prof. Bernard Asselain (methodologist and biostatistician), Prof. Catherine Paugam-Burtz (anaesthesiologist and intensive care physician), Prof. Samir Jaber (anaesthesiologist and intensive care physician) and Prof. Boris Jung (intensive care physician).

The DSMB, independent from the study sponsor and principal investigator, including three intensive care physicians, one methodologist and one drug safety specialist, will meet after inclusion of the first 20 patients to assess the safety of dornase alfa administration in ventilated trauma patients. The safety variables under study are detailed in the “Interventions” section. The DSMB will meet subsequently after further incremental inclusions of 100 patients. The DSMB charter was signed by all of its members.

#### Adverse event reporting and harms {22}

Adverse events and unintended effects of the trial intervention or trial conduct will be declared to the promotor within 24 h of occurrence. Moreover, the DSMB will meet after inclusion of the first 20 patients to assess the safety of dornase alfa administration in ventilated trauma patients. The safety variables under study are detailed in the following. For safety purposes, patient variables will be closely monitored before, during and within the first post-administration hour: lowest SpO_2_, maximal value and maximal increase in peak inspiratory airway pressure, maximal value and maximal increase in plateau airway pressure, extreme values of heart rate and mean arterial pressure, skin erythema, urticaria and variations in central temperature exceeding 1 °C. The DSMB will meet subsequently after further incremental inclusions of 100 patients.

#### Frequency and plans for auditing trial conduct {23}

In every centre, an audit will be performed by the Direction de la Recherche Clinique des Hôpitaux Universitaires de Strasbourg after inclusion of the first patient, then yearly and after enrolment of the last patient.

#### Plans for communicating important protocol amendments to relevant parties (e.g. trial participants, ethical committees) {25}

Important protocol modifications will be communicated to investigators, IRB and trial registries via e-mail. Every protocol amendment will be first submitted to the IRB and, after validation, transmitted to investigating centres, which will acknowledge receipt.

### Dissemination plans {31a}

The results of the study will be released to the participating physicians, referring physicians and medical community no later than 1 year after the completion of the trial, through presentation at scientific conferences and publication in peer-reviewed journals. Eligible authors will meet all four requirements of the ICMJE guidelines:

- Substantial contributions to the conception or design of the work; or the acquisition, analysis or interpretation of data for the work
- Drafting the work or revising it critically for important intellectual content
- Final approval of the version to be published
- Agreement to be accountable for all aspects of the work in ensuring that questions related to the accuracy or integrity of any part of the work are appropriately investigated and resolved

## Discussion

To the best of our knowledge, TRAUMADORNASE is the first large-scale study to evaluate the usefulness of inhaled dornase alfa to reduce the incidence of moderate-to-severe hypoxaemia in a population of severe trauma patients, who will also benefit from other lung-protective measures. The benefits are expected to include a reduction in both duration of mechanical ventilation and stay in the ICU, lower costs of hospital stay, fewer days on mechanical ventilation and a reduction in the selective pressure on multidrug-resistant bacteria.

In order to keep management practices as standardized as possible, we decided to limit the number of investigating centres to seven university-affiliated and non university-affiliated hospitals, all of which are recognized in the field of trauma care and treat more than 50 severe trauma patients per year. These centres belong to the TraumaBase network (www.traumabase.eu), which promotes multicentre clinical research on trauma and ensures consistent recording of clinical data according to the TraumaBase registry guidelines. These seven centres also share the same standards of care and, except for Pitié-Salpêtrière centre, belong to the same region of France (Grand Est).

From a translational point of view, the study will challenge the hypothesis that breaking up the double-stranded DNA backbone of both DAMPs and NETs with dornase alfa may reduce inflammation and NET-induced epithelial and endothelial cell injuries in the lungs of trauma patients.

Dornase alfa is a long-standingFDA-approved mucolytic agent used in cystic fibrosis patients. Its safety profile and limited side effects make it an appropriate candidate to curb DAMP-induced, NET-mediated inflammation. As we will use high-end vibrating mesh nebulizers, which provide excellent lung deposition and drug bioavailability, we expect that dornase alfa will be deposited within the depth of the lung parenchyma, where it may be the most useful.

### Study limitations

The incidence of moderate-to-severe hypoxaemia is the primary study endpoint. A 45% basal incidence of moderate-to-severe hypoxaemia may appear overstated to some experts, but it must be underlined that only severe trauma patients will be included and that a 45% incidence was reported in the last randomized PROMTT trial [13], in the era of damage-control resuscitation [54]. A 15% absolute reduction seems ambitious for a single intervention. However, previous studies using dornase alfa in animal lung injury models and in ventilated patients suffering atelectasis demonstrated striking results [35, 38–41, 55].

Because fluid loading regimens and transfusion strategies are based on local written protocols, they may act as potential confounding variables. However, this will be controlled by the stratification of the randomization at the centre level and adjustment of statistical analyses in cases of differences between groups.

In conclusion, this trial is the first multicentre, randomized controlled, double-blinded study adequately powered to test the hypothesis that aerosolized dornase alfa reduces the incidence of moderate-to-severe hypoxaemia in mechanically ventilated severe trauma patients.

## Trial status

Protocol version 8.0 was approved by the National Institutional Review Board on 6 November 2018. The study started on 10 March 2019 and is expected to last until September 2022 (36-month inclusion period plus 6-month participation period).

After validation from its Scientific Committee, the TRAUMADORNASE study was funded by the French Ministry of Health.

## Data Availability

Only the principal investigators, the DSMB and the statisticians will have access to the final data set. The data sets used and analysed during the current study will be available from the corresponding author on reasonable request, after publication of the main core article.

## Abbreviations

ARDS: Acute respiratory distress syndrome
ATS: American Thoracic Society
CDC: Center for Disease Control and Prevention
CPOT: Critical Care Pain Observation Tool
DALY: Disability-adjusted life year
DAMP: Damage-associated molecular pattern
DSMB: Data safety and monitoring board
EDTA: Ethylenediaminetetraacetic acid
ELISA: Enzyme-linked immunosorbent assay
FDA: US Food and Drug Administration
FiO_2_: Inspired oxygen fraction
HMGB1: High mobility group box-1
HSP: Heat shock proteins
ICMJE: International Committee of Medical Journal Editors
ICU: Intensive care unit
IRB: Institutional review board
ISS: Injury Severity Score
McMC: Markov chain Monte Carlo
MOF: Multi-organ failure
MRC: Medical Research Council
NE: Neutrophil elastase
NET: Neutrophil extracellular trap
PaO_2_: Partial arterial oxygen tension
PEEP: Positive end-expiratory pressure
qPCR: Quantitative polymerase chain reaction
RAGE: Receptors for advanced glycation end products
RASS: Richmond Agitation and Sedation Scale
SOFA: Sequential Organ Failure Assessment
SSL: Secure Sockets Layer
SSVS: Stochastic search variable selection
TLR: Toll-like receptor
VAP: Ventilator-associated pneumonia
